# Heterotopic ossification in the reaming tract of a percutaneous antegrade femoral nail: a case report

**DOI:** 10.1186/1752-1947-7-90

**Published:** 2013-04-04

**Authors:** Sergiu Botolin, Cyril Mauffrey, E Mark Hammerberg, David J Hak, Philip F Stahel

**Affiliations:** 1 Department of Orthopaedic Surgery, Denver Health Medical Center, University of Colorado, School of Medicine, 777 Bannock Street, Denver, CO, 80204, USA

**Keywords:** Heterotopic ossification, Femoral nailing, Femur fracture, Complication

## Abstract

**Introduction:**

Heterotopic ossification is a rare complication of musculoskeletal injuries, characterized by bone growth in soft tissues. Percutaneous antegrade intramedullary nailing represents the ‘gold standard’ for the treatment of femur shaft fractures. Minor bone growth is frequently seen around the proximal end of reamed femoral nails (so-called ‘callus caps’), which are asymptomatic and lack a therapeutic implication. The occurrence of excessive, symptomatic heterotopic ossification around the entry site of an antegrade femoral nail is rarely described in the literature.

**Case presentation:**

We present the case of a 28-year-old Caucasian woman who developed extensive heterotopic ossification around the reaming seeds of a reamed femoral nail. She developed severe pain and significantly impaired range of motion of the hip joint, requiring revision surgery for heterotopic ossification resection and adjunctive local irradiation. She recovered full function of the hip and remained asymptomatic at her two-year follow-up appointment.

**Conclusions:**

Severe heterotopic ossification represents a rare but potentially detrimental complication after percutaneous femoral nailing of femur shaft fractures. Diligent care during the reaming procedure, including placement of a trocar to protect from osteogenic seeding of the soft tissues, may help decrease the risk of developing heterotopic ossification after reamed antegrade femoral nailing.

## Introduction

Heterotopic ossification (HO) represents a debilitating condition characterized by bone formation in soft tissue that normally does not calcify [1-3]. HO occurs mainly around the hip and elbow joint, and is associated with subjective pain and progressive joint stiffness [4,5]. The pioneer of intramedullary nailing, Gerhard Küntscher, was among the first to report ‘callus caps’ in the abductor region of the hip after femoral nail fixation in the 1960s [6]. Subsequent studies in the 1990s revealed that the incidence of HO was significantly decreased in unreamed compared to reamed femoral nailing [7]. However, due to its biological and mechanical superiority, reamed antegrade intramedullary nailing (IMN) remains the accepted ‘gold standard’ for the management of femur shaft fractures [8]. This case report presents the rare scenario of a young patient who developed significant HO at the entry site of an interlocking femoral nail after percutaneous antegrade reamed IMN fixation of a femoral shaft fracture. Presumptive root causes, preventability, and therapeutic measures are discussed and placed into perspective of the pertinent peer-reviewed literature.

## Case presentation

**Figure 1:**
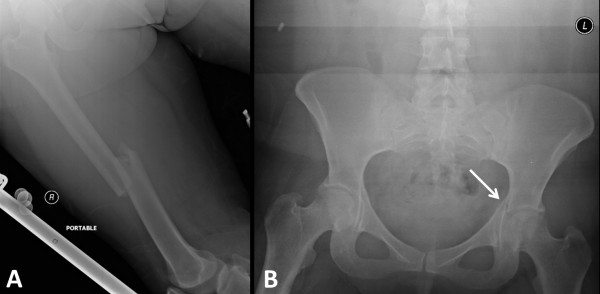
Initial radiographic workup of a 28-year-old woman involved in a high-speed motor vehicle accident, sustaining a right femoral shaft fracture (A) and a contralateral nondisplaced associated transverse/posterior wall acetabular fracture (arrow in B).

**Figure 2:**
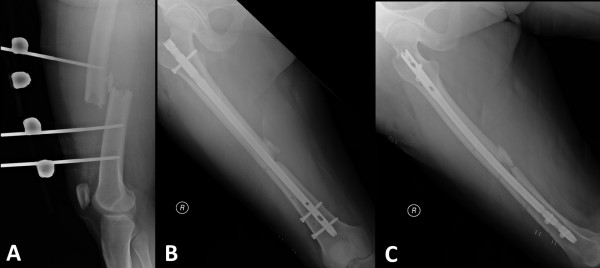
Acute management of the femur shaft fracture by temporary ‘damage control’ external fixation (A) and scheduled conversion to an antegrade reamed intramedullary interlocking nail (B,C) after the patient had been fully resuscitated.

A 28-year-old Caucasian woman was an unrestrained passenger in a high-speed motor vehicle collision. She sustained a blunt chest trauma, a right closed transverse mid-shaft femur fracture (Figure 1A), and a contralateral left nondisplaced associated transverse/posterior wall acetabular fracture (Figure 1B, arrow). The patient had a Glasgow Coma Scale (GCS) score of 15 on initial presentation, with no evidence of head or spine trauma. Due to the high-energy mechanism and borderline hemodynamic stability, with a lactate level of 3.3mmol/L and base deficit of −8mEq/L, a decision was made for temporary ‘damage control’ external fixation (Figure 2A), followed by an uneventful conversion to IMN fixation after full resuscitation three days later (Figure 2B,C), using a percutaneous technique [9] with a cannulated titanium interlocking nail (Synthes, Paoli, PA, USA). There were no signs of a soft-tissue degloving injury (Morel-Lavallée lesion) around the ipsilateral hip and thigh. The patient had a body mass index (BMI) within a normal range (21.6kg/m^2^). The procedure was performed uneventfully and without intraoperative complications by a fellowship-trained orthopaedic trauma surgeon. The piriformis fossa starting point was easily identified intraoperatively in a standard fashion under fluoroscopic guidance, without excessive attempts for identifying the ‘perfect’ starting point. The length of the percutaneous incision was 2cm, as previously described [9]. The IMN entry point at the proximal femur was reamed without the use of a trocar for soft-tissue protection.

**Figure 3:**
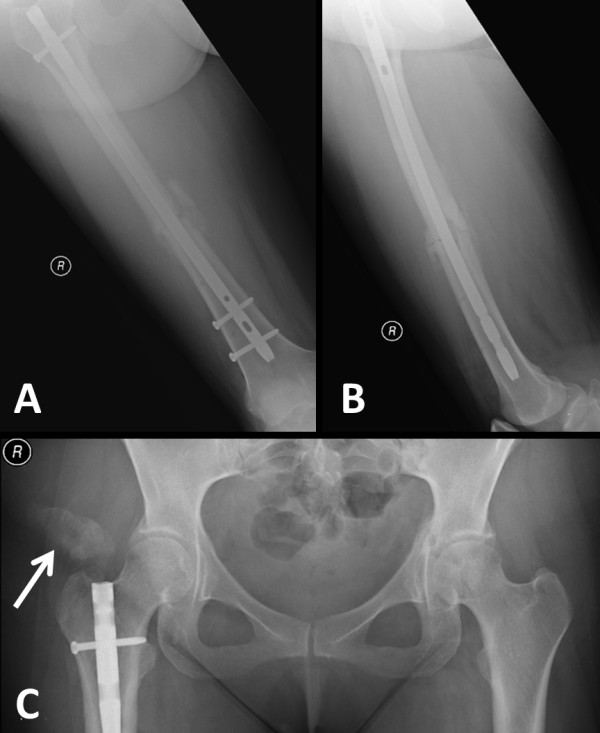
**Evidence of fracture healing of the right femur fracture (A,B) and the nonoperative contralateral acetabular fracture (C) at two months of follow-up.** The pelvic X-ray reveals early signs of heterotopic ossification in the soft tissues at the entry site of the femoral nail (arrow in **C**).

**Figure 4:**
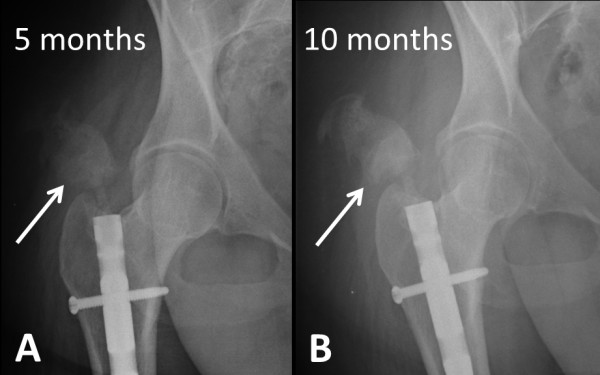
Progressive extent of heterotopic ossification around the right hip is seen tracking down to the entry point of the femoral nail on follow-up X-rays at five months (arrow in A) and 10 months (arrow in B).

**Figure 5:**
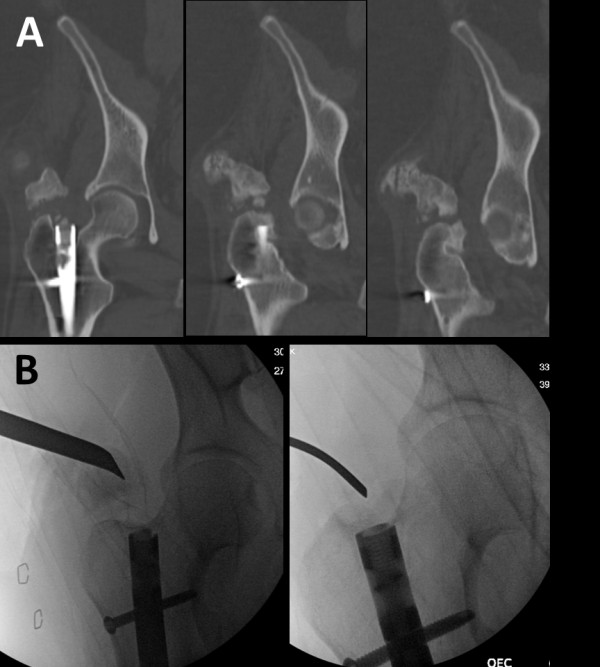
Preoperative computed tomography (CT) scan (A) and intraoperative fluoroscopy images during the surgical resection procedure (B).

**Figure 6:**
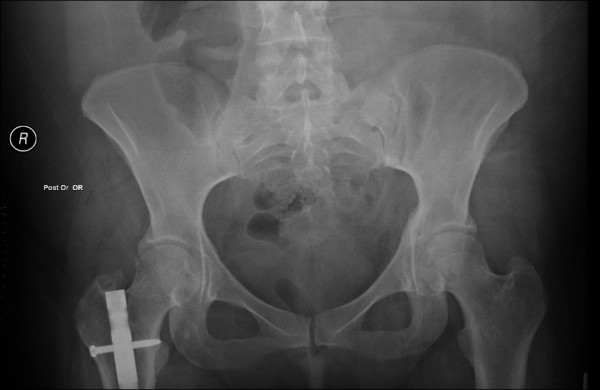
A postoperative pelvic radiograph documents the successful resection of the heterotopic ossification around the right hip.

**Figure 7:**
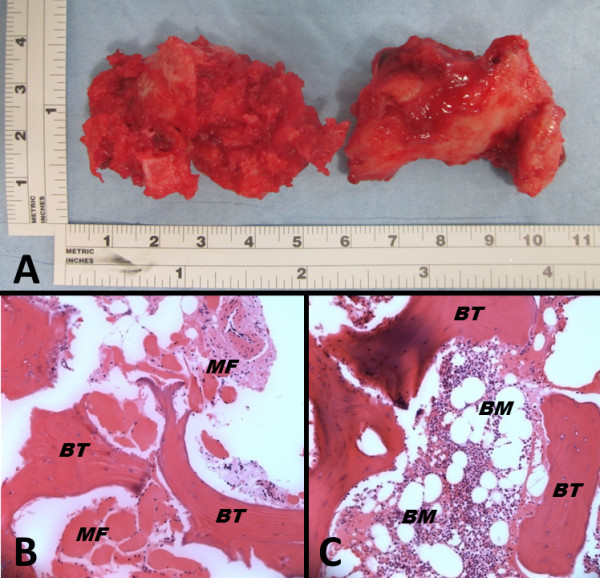
**Two major bone masses were surgically excised (A). **Histopathology confirmed the classic features of heterotopic ossification **(B,C)**. BM, bone marrow; BT, bony trabecula; MF, muscle fibers.

After surgery, the patient was mobilized with full weight-bearing on the right side (femur fracture), and with touch-down weight-bearing on the left side (nonoperative acetabular fracture). She was placed on low-molecular-weight heparin (dalteparin) for venous thromboembolism prophylaxis and discharged home after clearance by physical therapy on hospital day five. The patient followed up in the outpatient clinic for a wound check and staple removal at two weeks, and for radiographic assessment of fracture healing at two months (Figure 3). At that point in time, the patient had minimal complaints, and radiographs revealed uneventful healing of the right femur fracture (Figure 3A,B) and the left acetabular fracture (Figure 3C). Her pelvic X-ray revealed early signs of HO formation at the entry point of the antegrade femoral nail (Figure 3C, arrow), but the patient appeared to be asymptomatic with regard to her right hip function. The patient was seen again five months after surgery. At that time, her chief complaint consisted of right hip pain at the incision site of the nail insertion, with a solid palpable mass in the deep soft tissue layer, which was tender on palpation. She claimed that the pain had drastically limited her ambulation, and that she was unable to abduct the hip. Radiographs demonstrated increasing HO formation over the right hip, tracking to the piriformis fossa insertion site of the femoral nail (Figure 4A, arrow). The implications of HO were discussed in-depth with the patient, including the recommendation to allow for the HO activity to ‘burn out’, and to follow up with a formal discussion on surgical resection options in a delayed fashion. At 10 months, the patient returned to the clinic reporting continued and increasing pain in her right hip, with a progressive inability to move the hip. The clinical evaluation revealed the following passive range of motion of the right hip: internal rotation (10°), external rotation (30°), flexion (100°), extension (10°), abduction (0°), adduction (20°). Radiographs confirmed the notion of increased HO formation over the right hip (Figure 4B, arrow). A preoperative bone scan was obtained, which demonstrated the absence of activity within the heterotopic bone formation (not shown). After extensive discussion of the risks and benefits of surgical HO resection and concomitant peri-operative radiotherapy for attenuating the risk of HO recurrence, the patient agreed to proceed with the procedure as discussed. A computed tomography (CT) scan of the right hip was obtained for preoperative planning (Figure 5A), and a complete resection of the bone mass in the abductors of the hip was successfully and safely achieved through a modified Hardinge approach (Figure 5B and Figure 6). The fragments of the excised bone mass are shown in Figure 7A. The diagnostic workup by histopathology confirmed microscopic findings consistent with heterotopic ossification (Figure 7B,C). The patient tolerated the surgical procedure well. She underwent a single radiation therapy of 7Gy to the operative site on postoperative day one, and was started on indomethacin 25mg orally three times a day for a total of six weeks. The rationale for selecting a combination therapy of radiation and indomethacin was based on recent literature, which appears to favor the combination therapy as being superior in preventing HO recurrence [10,11]. Her symptoms had completely resolved at the first follow-up appointment at two weeks for wound check and staple removal. At that point in time, her postoperative passive range of motion of the right hip had significantly improved: internal rotation (30°), external rotation (40°), flexion (120°), extension (10°), abduction (45°), adduction (30°). The patient had an uneventful further recovery with excellent subjective and functional long-term outcome at two years after the initial index procedure.

## Discussion

Heterotopic ossification (HO) after antegrade reamed femoral nailing is a common postoperative problem that, however, frequently lacks a therapeutic implication [6,7,12]. Brumback and colleagues implemented a classification system of HO after femoral intramedullary nailing based on the length of the HO, which is the distance between the proximal end of the nail and the most proximal end of the HO [12]. This classification is based on increasing severity from grade 0 to IV [12]. The patient presented in the current report developed HO grade III (that is >2cm but without extension to the pelvis). Biyani and colleagues investigated hip abduction in patient with HO and found marked hip abductor weakness of 32 to 80 percent (mean 48.6 percent) in patients with various degrees of HO [13]. Our patient developed a complete impairment of hip abduction (0°) that was restored to a normal function after HO excision. Although HO in our patient did not induce a complete ankylosis of the hip joint, surgical excision demonstrated two large HOs measuring more than 4cm in length (Figure 7A) that together drastically affected hip function, to a limited range of motion described in detail above. As supported by the successful outcome in the present study, we recommend considering HO resection, in conjunction with peri-operative irradiation, at a time point when the activity of the heterotopic ossification appears resolved on a preoperative bone scan.

Ongoing debate as far back as the 1980s centered on the notion that small incisions are not necessarily associated with a decreased extent of soft tissue trauma [6], a problem which may directly relate to the operative technique applied in the present case [9]. Multiple root causes of HO induced after reamed femoral nailing have been discussed in the literature, including the reasonable argument of local seeding of osteogenic reaming debris in the surrounding soft tissues [8]. However, most available data are derived from experimental animal studies [14,15]. In support of the notion of osteogenic reaming debris contributing to the development of HO, Furlong *et al*. demonstrated a significant decrease in HO incidence with nonreamed femoral nailing compared to traditionally reamed IMN (9.4 percent versus 35.7 percent) [7]. However, the residual incidence of almost 10 percent of HO in nonreamed IMN provides an argument against the reaming debris as the sole contributing root cause [7]. Since reamed femoral IMN represents the current ‘gold standard’ for fixation of femur shaft fractures [8,9], a deviation from this concept would likely increase the risk for complications related to unreamed nailing, including hardware failure, malunion, and nonunion [16,17].

Intuitively, one would argue that copious soft tissue irrigation prior to wound closure would decrease the risk of HO by washing out the osteogenic reaming debris. However, the study by Brumback and colleagues failed to support this notion, since a standardized wound irrigation with 3L of saline by pulsatile lavage did not decrease the incidence of HO [12]. Ischemic injury to the hip abductors represents a known root cause of HO, which may be minimized by diligent care of the soft tissues during the femoral nailing procedure [2]. Based on the currently available evidence, it is fair to conclude that both the reaming debris and the extent of traumatic and intraoperative injury to the surrounding soft tissues at the operative site play a role in the development of HO after antegrade reamed femoral IMN. The management of symptomatic HO is purely surgical, whereas, the established secondary prophylaxis consists of nonsteroidal anti-inflammatory drugs (NSAIDs) (such as indomethacin), in conjunction with preoperative or immediate postoperative local irradiation of the operative site [18]. This concept was successfully applied in the patient presented in the current case report, and the patient was asymptomatic at two years of follow-up after the index procedure of femoral nailing.

## Conclusions

Symptomatic HO after reamed IMN of femur shaft fractures represents a common postoperative complication, albeit without clinical relevance in most cases. As outlined in the present case report, patients may rarely develop significant HO characterized by subjective pain and compromised hip function, up to full joint stiffness (ankylosis). Osteogenic reaming debris and operative soft-tissue injury appear to represent the main known root causes of HO after femoral nailing. Despite the absence of strong evidence, the diligent intraoperative care of the soft tissues in conjunction with the use of a trocar during the reaming process to avoid local seeding of reaming debris, and copious fluid irrigation, appear to represent intuitive parameters that can be influenced by the surgeon. Further research is required to fully understand the pathogenesis of HO and to determine risk factors, root causes, and preventability of this potentially detrimental complication.

## Consent

Written informed consent was obtained from the patient for publication of this case report and accompanying images. A copy of the written consent is available for review by the Editor-in-Chief of this journal.

## Competing interests

Drs Hak, Hammerberg, and Stahel have received occasional speaker’s honoraria from Synthes (Paoli, PA, USA) in the past five years. The authors declare no other financial competing interests related to this case report.

## Authors’ contributions

SB drafted the first version of this manuscript. PFS performed the patient’s revision surgery, designed the case report, and revised the initial draft of the paper. CM, EMH, and DJH wrote the discussion and significantly contributed to the critical revisions of the final manuscript. All authors read and approved the final version prior to submission.
